# Plasticity in the Oxidative Folding Pathway of the High Affinity *Nerita Versicolor* Carboxypeptidase Inhibitor (NvCI)

**DOI:** 10.1038/s41598-017-05657-7

**Published:** 2017-07-14

**Authors:** Sebastián A. Esperante, Giovanni Covaleda, Sebastián A. Trejo, Sílvia Bronsoms, Francesc X. Aviles, Salvador Ventura

**Affiliations:** 1 Institut de Biotecnologia i de Biomedicina and Departament de Bioquímica i de Biologia Molecular, Universitat Autònoma de Barcelona, 08193 Bellaterra, (Barcelona) Spain; 2 Servei de Proteòmica i Biologia Estructural, Universitat Autònoma de Barcelona, Barcelona, Spain; 3 Instituto Multidisciplinario de Biología Celular (IMBICE) – CONICET, La Plata, Argentina

## Abstract

*Nerita Versicolor* carboxypeptidase inhibitor (NvCI) is the strongest inhibitor reported so far for the M14A subfamily of carboxypeptidases. It comprises 53 residues and a protein fold composed of a two-stranded antiparallel β sheet connected by three loops and stabilized by three disulfide bridges. Here we report the oxidative folding and reductive unfolding pathways of NvCI. Much debate has gone on whether protein conformational folding guides disulfide bond formation or instead they are disulfide bonds that favour the arrangement of local or global structural elements. We show here that for NvCI both possibilities apply. Under physiological conditions, this protein folds trough a funnelled pathway involving a network of kinetically connected native-like intermediates, all sharing the disulfide bond connecting the two β-strands. In contrast, under denaturing conditions, the folding of NvCI is under thermodynamic control and follows a “trial and error” mechanism, in which an initial quasi-stochastic population of intermediates rearrange their disulfide bonds to attain the stable native topology. Despite their striking mechanistic differences, the efficiency of both folding routes is similar. The present study illustrates thus a surprising plasticity in the folding of this extremely stable small disulfide-rich inhibitor and provides the basis for its redesign for biomedical applications.

## Introduction

Deciphering how a string of amino acid residues folds into a biologically active three-dimensional structure remains a major challenge in structural biology^1–7^. Much effort has gone into identifying the productive intermediates that are assumed to be necessary for rapid protein folding^4, 5, 8–10^. However, folding intermediates are usually difficult to isolate and characterize due to their short half-life and highly flexible structures.

The formation of disulfide bonds adds an additional layer of complexity to the folding pathway of many proteins. The term ‘oxidative folding’ describes the composite process by which a reduced, unfolded protein gains both its native disulfide bonds (disulfide-bond formation) and its native structure (conformational folding)^11, 12^. Disulfide bonds display unique chemical and structural characteristics that allow them to be used as probes to monitor the progression of protein folding/unfolding pathways and of the structural properties of the intermediates^11, 13^. Indeed, the landmark discovery that the information to fold a protein is fully contained in the primary amino acid sequence was based on oxidative folding experiments with RNase A, a protein containing four disulfide bonds^14^.

Oxidative folding is a very complex process, where an interplay between conformational folding, disulfide bond formation and disulfide isomerization takes place^15, 16^. The relationship between these three reactions is not trivial, since oxidation, reduction and reshuffling rates are modulated by the effective concentration of thiolate anions as well as by the reactivity, proximity and accessibility of both free cysteines and disulfide bonds, which, in turn, depend on their relative burial in the intermediate conformations during folding^11, 12, 16–18^. The formation of native disulfide bonds can promote and accelerate the arrangement of local or global secondary and tertiary structure elements or, conversely, strong structural propensities might force the preferential formation of native disulfide bonds^12^. Indeed, whether disulfide bond formation drives protein folding or vice versa, has remained a major unsolved problem in protein folding, despite its chemical and biophysical relevance^19^.

The analysis of oxidative folding reactions, pioneered by Creighton^20^, is based in the slow kinetics and particular chemistry of disulfide bond formation. By using an appropriate thiol quenching reaction, such as alkylation or acidification, disulfide bond formation can be stopped and disulfide intermediates can be isolated. This approach has been applied for the elucidation of the folding pathways of more than 30 different disulfide-rich proteins (reviewed in refs 21–23). To date, these studies have revealed a great diversity of folding pathways that differ mainly in the heterogeneity and native disulfide-bond content of the intermediates that accumulate along the course of oxidative folding. Two extreme opposite models of oxidative folding are exemplified by bovine pancreatic trypsin inhibitor (BPTI) and hirudin. The BPTI-like model is characterized by the predominance of a limited number of intermediates that adopt native disulfide bonds and native-like substructures^24^. Conversely, the hirudin-like model is defined by a highly heterogeneous population of intermediates containing mostly non-native disulfides, including the presence of scrambled isomers (fully oxidized species that contain at least two non-native disulfide bonds)^25^. Both BPTI and hirudin are protease inhibitors. Proteins displaying this activity usually comprise small folds cross-linked by several disulfides and accordingly have been among the most valuable models to dissect oxidative folding reactions (reviewed in ref. 26).

Metallocarboxypeptidases (MCPs) are zinc-dependent enzymes that hydrolyse the C-terminal residue(s) of peptides and proteins. Their activity is tightly regulated and normally takes place outside of the cell. Mammals contain several types of MCPs that are involved in a great variety of physiological processes, from digestion, to blood coagulation/fibrinolysis, tissue organogenesis, neurohormone and cytokine maturation processing, among others. Several MCPs have been linked to human diseases, such as cancer, alzheimer disease, type 2 diabetes, acute pancreatitis and pancreas cancer, fibrinolysis and inflammation. Consequently, MCPs and their strong, small protein inhibitors have been of considerable medical interest as potential drug targets or effectors^27, 28^. The study of folding/unfolding pathways allows to gain insight into the essential elements that confer foldability and stability to MCPs inhibitors and is prerequisite for their production, minimization and redesign for biomedical and biotechnological purposes.

**Figure 1:**
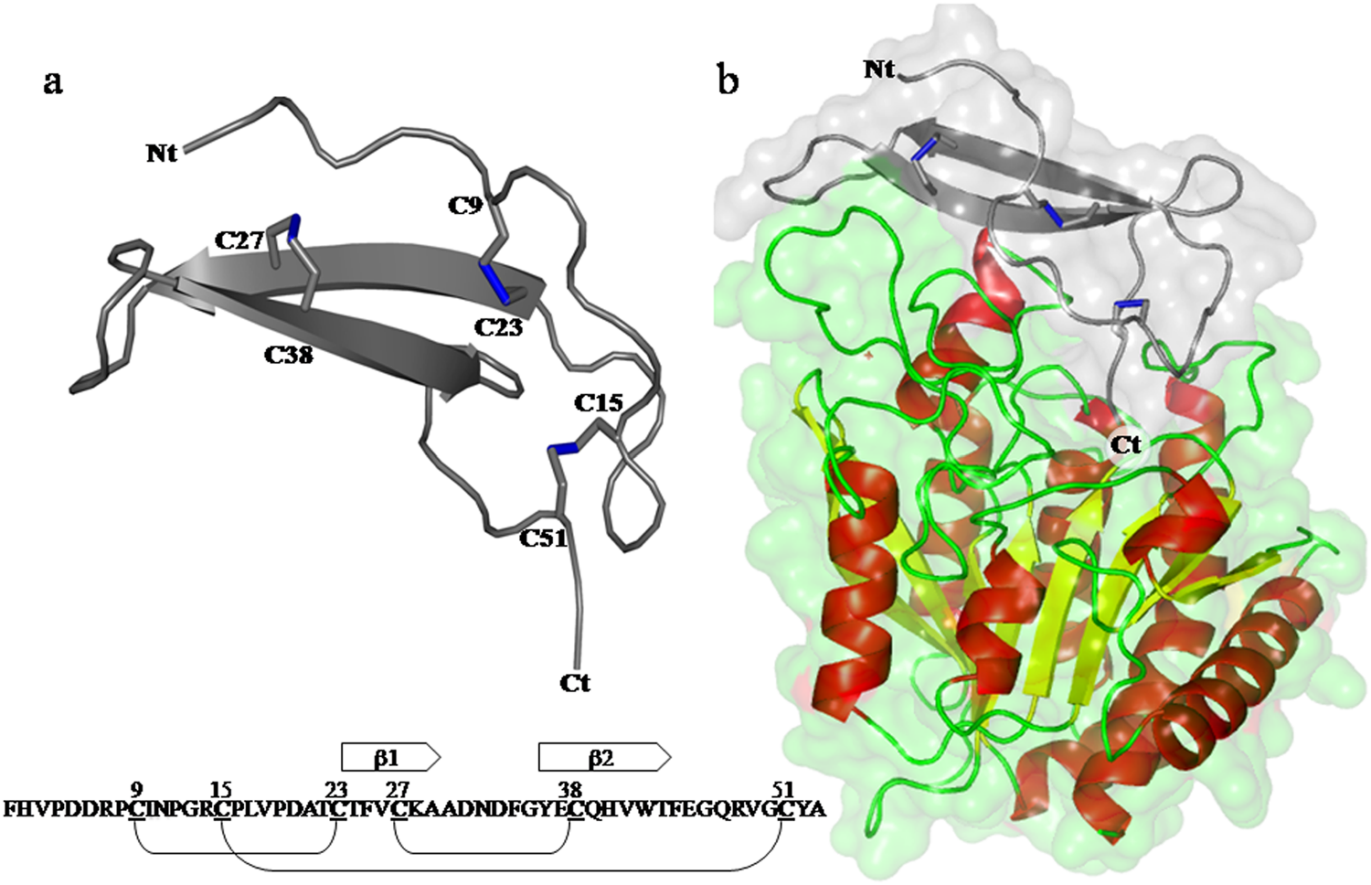
Schematic view of the native three-dimensional structure of NvCI and the NvCI-hCPA4 complex. (**a**) Schematic view of the ribbon representation of NvCI. The cysteine residues are depicted in the structure and the disulfide bonds are shown in stick representation (*blue*). The amino acid sequence of NvCI and its secondary structure elements and disulfide pairing are schematically shown at the bottom. The inhibitory site for MCPs comprises two residues (Tyr52 and Ala53), located at the C terminal tail, after Cys51. (**b**) Surface and ribbon representation of human CPA4 (hCPA4) in complex with NvCI (*gray*). The three disulfide bridges formed in NvCI are shown in stick representation (*blue*). The α-helix, β-strands, and coils of hCPA4 are highlighted in *red*, *yellow*, and *green* color, respectively. Ct and Nt stands for C terminus and N terminus, respectively. The Protein Data Bank accession number for the structure of NvCI in complex with hCPA4 is 4A94. All figures were prepared with PyMOL.

*Nerita versicolor* carboxypeptidase inhibitor (NvCI) is the strongest, natural proteinaceous inhibitor reported so far for the M14A subfamily of carboxypetidases, with inhibitory constants in the picomolar range^29^. It comprises 53 residues and a protein fold composed of a two-stranded antiparallel β-sheet connected by three loops and stabilized by three disulfide bridges: The Cys27-Cys38 disulfide bond interconnects the two major secondary structure elements; Cys9-Cys23 stabilizes the first beta sheet by fixing this element to the N terminus of the inhibitor and Cys15-Cys51 links the C terminal tail, which corresponds to the active site of NvCI, with the first loop (Fig. 1). This small protein was isolated from the marine snail *Nerita versicolor* and represents the first proteinaceou*s* inhibitor of MCPs isolated and characterized in depth from a marine organism^29^. Other proteinaceous carboxypeptidase inhibitors have been found in evolutionarily distant organisms such as potato (Potato carboxypeptidase inhibitor: PCI)^30^, the medical leech *Hirudo medicinalis* (leech carboxypeptidase inhibitor: LCI)^31^, the tick *Rhipicephalus bursa* (tick carboxypeptidase inhibitor: TCI)^32^ and the intestinal parasite *Ascaris Suum* (Ascaris carboxypeptidase Inhibitor: ACI)^33, 34^. The three-dimensional protein structures of these inhibitors are unrelated and completely different^26^, the only conserved motif being the structural conformation of the P1 and P2 residues in the C-terminal tail of the inhibitor that interacts in a competitive manner with the active site of the carboxypeptidase by occlusion of the active site subsites S1’, S1 and S2^35^.

Here, we report a comprehensive analysis of the pathways of oxidative folding and reductive unfolding of NvCI. The characterization of the acid-trapped intermediates that accumulate along these two processes reveals the predominance of a few native disulfide-bonded intermediates that efficiently guide this protein toward its native conformation. These results identify NvCI as a protein with a BPTI-like oxidative folding under native conditions. However, when non-covalent interactions are nullified by the presence of denaturants, the protein is still able to fold efficiently through a “trial an error” mechanism resembling that of hirudin. Therefore, NvCI arises as a novel model to study the inherent plasticity of protein folding pathways.

## Results

### Oxidative folding of NvCI

**Figure 2:**
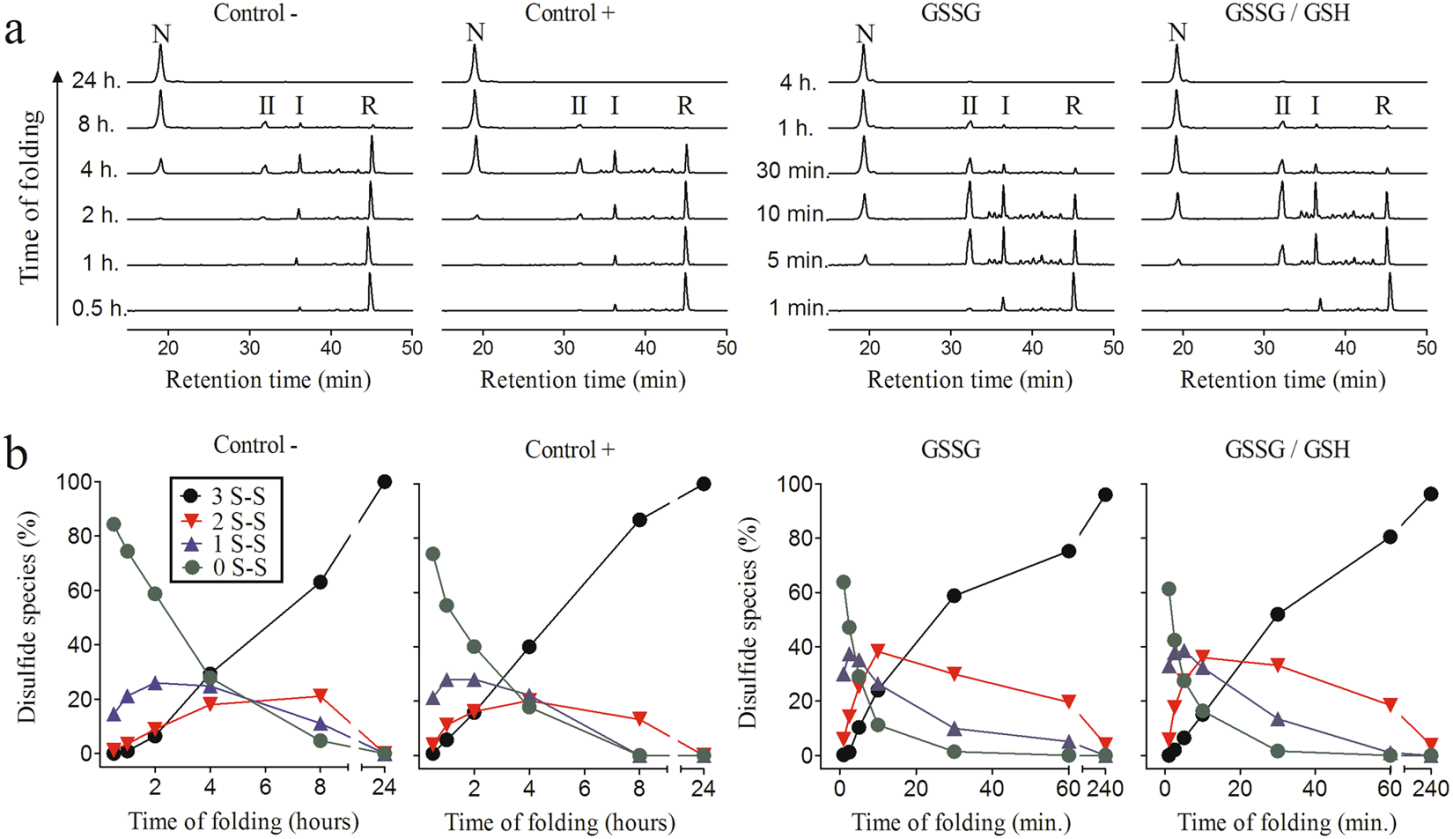
RP-HPLC analysis of the folding intermediates and quantitative analysis of disulfide species along the oxidative folding pathway of NvCI. Folding was carried out in Tris-HCl buffer (pH 8.4) in the absence (Control−) and presence of selected redox agents: 0.25 mM 2-mercaptoethanol (Control+), 0.5 mM GSSG or a mixture of 0.5 mM GSSG and 1 mM GSH. (**a**) Chromatographic profiles of the folding reactions. Intermediates were acid-trapped at the noted times and analyzed by RP-HPLC. Retention times of the native (N) and fully reduced/unfolded (R) forms as well as the two major intermediates (I and II) are indicated. (**b**) Disulfide species in the oxidative folding of NvCI. Intermediates were trapped by alkylation of the free cysteines at various times and analyzed by MALDI-TOF-MS. 0S-S, 1S-S, 2S-S, and 3S-S represent the completely reduced, one disulfide, two disulfide, and three disulfide species, respectively.

Reduced and unfolded NvCI was allowed to refold in Tris-HCl buffer (pH 8.4), in the absence and presence of different redox agents. Folding intermediates were trapped at selected time points by either acid quenching with TFA or alkylation of the free SH groups. The heterogeneity and chromatographic behavior of the acid-trapped folding intermediates was characterized by RP-HPLC (Fig. 2a) and the disulfide bond content of the derivatized intermediates was determined by MALDI-TOF-MS analysis (Fig. 2b). In the absence of redox agents (control−), only few intermediates populate the folding reaction, with the accumulation of two major fractions, designated as I and II (Fig. 2a). The presence of 0.25 mM 2-mercaptoethanol (control+), which promotes disulfide rearrangement in oxidized species, did not affect the overall chromatographic pattern. However, if we compare the 4 h acid-trapped sample in both conditions, the recovery of native protein was higher in control+, indicating that the folding rate was slightly increased. Taking into account that NvCI belongs to the usually slow-folding small disulfide-rich protein class, its overall folding process was very efficient, allowing an almost complete recovery of the native protein after 8 hours of refolding both in the absence and presence of redox agent. Mass spectrometry analysis of disulfide species along NvCI folding reaction showed the formation of 1-disulfide species (1S-S) within the first 4 hours of refolding, to reach a maximum around 2 to 3 hours (Fig. 2b). At this time point, an increase in 2-disulfide species (2S-S) started, reaching a maximum at about 6 to 8 hours, to finally convert into the 3-disulfide native end product (3S-S). Oxidative folding experiments are usually performed at pH 8.4, because this is the pKa of the Cys amino acid side chain. In order to confirm that the protein folds through the same pathway at physiological pH, the refolding experiments were also performed in 0.1 M Tris at pH 7.4 both in the absence and presence of 2-mercaptoethanol (Supplementary Fig. S1a). In these conditions, we detect exactly the same folding intermediates than at pH 8.4; despite, as expected, the folding reaction evolves slightly more slowly.

Oxidative folding of NvCI was subsequently performed in the presence of oxidized glutathione (GSSG) (0.5 mM) and a mixture of reduced and oxidized glutathione (GSH:GSSG, 1.0:0.5 mM, respectively). The RP-HPLC patterns obtained in both redox conditions were almost indistinguishable and the folding reaction progressed at very similar rates, indicating that the presence of the reducing agent (GSH) did not have a significant effect on NvCI folding (Fig. 2a). Consequently, the strong differences observed between the control experiment and those performed under redox conditions, could be attributed mostly to the oxidizing agent (GSSG). The presence of GSSG affected both the overall folding rate and the isoforms pattern. Native NvCI was almost fully formed after 1 hour, compared to the 8 hours required in the control experiment. Besides, folding intermediates I and II accumulated to a much greater extent and other minor intermediate species become detectable under these conditions. The fast oxidation of 1- and 2-disulfide species resulted in their accumulation, as it can be observed in the 10-min refolding time (Fig. 2b), when nearly 70% of the species are 1- or 2-disulfide forms, while in the control experiments the sum of these species hardly accounts for 40% of the conformers. In these oxidizing conditions intermediates I and II seem to behave as kinetic traps, their conversion becoming rate-limiting for the attainment of the native fold.

Protein folding reactions usually exhibit significant temperature dependence^36^. To test if this is the case for NvCI, we performed oxidative folding experiments on ice, in 0.1 M Tris.Cl pH 8.4, in the absence and presence of 0.25 mM 2-mercaptoethanol. The samples were acid quenched at different timepoints and subjected to RP-HPLC. As expected, the folding reaction rates were significantly lower in these conditions, with only 30–40% of the native protein being recovered after 24 h (Supplementary Fig. S1b). Nevertheless, the pathway remained identical to that recorded at 25 °C in terms of the succession of folding intermediates.

To assess the disulfide bond content of the two major folding intermediates, they were purified by RP-HPLC, freeze-dried and alkylated either with 4-vinylpyridine (VP) or iodoacetamide (IAA). The intermediate fractions I and II displayed 4 and 2 reduced reactive cysteines, respectively. Therefore, intermediate I contains a single disulfide bond while intermediate II contains two disulfide bonds. The intermediate species that accumulated to a lesser extent were also purified from the 10-min GSSG acid-trapped sample. That is, the fraction eluting between the reduced protein and peak I (retention time from 39 to 45 min) and the fraction eluting between peak I and II (retention time from 35.5 to 37 min. See Fig. 6a for a summary of RP-HPLC fractions and disulfide content). MS analysis of the former showed that it contained mainly one-disulfide species, while the latter contained mainly two-disulfide species. Importantly, none of the one- or two-disulfide species elutes close to native NvCI indicating that under chromatographic conditions they are much less compact than the natively cross-linked form. In addition, no alternative, scrambled, three-disulfide species populate the reaction in any of the assayed folding conditions.

**Figure 3:**
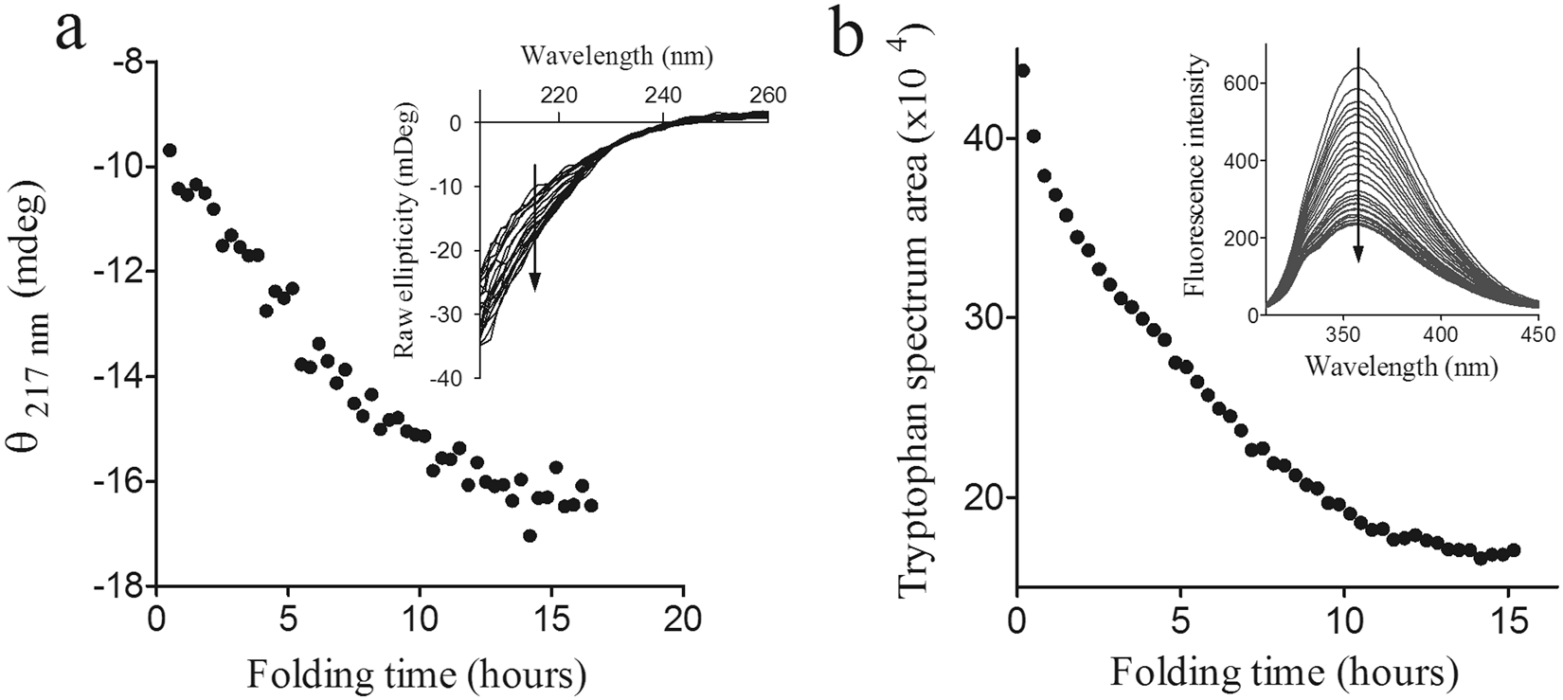
Secondary and tertiary structural changes upon oxidative folding of NvCI. The secondary and tertiary structure changes along folding time were monitored by far-UV CD and tryptophan fluorescence spectra measurements, respectively. The fully reduced/unfolded protein was allowed to refold in 0.1 M Tris-HCl pH 8.4 at 20 °C. (**a**) Plot of ellipticity at 217 nm as a function of folding time. Inset: Far-UV CD spectra at different time points along folding. (**b**) Tryptophan spectrum area changes as a function of folding time. Inset: Tryptophan fluorescence spectra at different time points along folding. The arrow indicates the progression of the folding reaction along time.

To further characterize the conformational changes of the inhibitor along the folding process, the fully reduced/unfolded NvCI was allowed to refold in Tris-HCl (pH 8, 4) and the secondary and tertiary structural changes were monitored using far-UV CD and tryptophan emission fluorescence spectroscopy, respectively (Fig. 3). The only secondary structure element in native NvCI protein is a two-stranded antiparallel β-sheet^29^. Accordingly, as the protein folds, the far-UV CD intensity at 217 nm increases, reaching a maximum after 10 h when all the protein is in the folded state (Fig. 3). NvCI harbours a single tryptophan at position 42 in the second β-strand (Fig. 1a). In the native state, Trp42 is buried inside NvCI, being packed between the Cys9-Cys23 and Cys15-Cys51 disulfide bonds, which likely contribute to the quenching of its fluorescence. As a consequence, the intrinsic fluorescence intensity of the native state is much lower than that of the reduced/unfolded state, despite not detecting any differences in the emission maximum wavelength. When the protein was allowed to refold in the same conditions described above, we observed a decrease in the Trp42 fluorescence intensity along folding that again became stabilized after 10 h (Fig. 3b). The spectroscopic probes suggested that the formation of the β-strand upon folding is coupled to the burial of Trp42 in the native structure. The kinetics of secondary and tertiary structure formation are consistent with the population of the native state as measured by RP-HPLC.

### Reductive unfolding of NvCI

**Figure 4:**
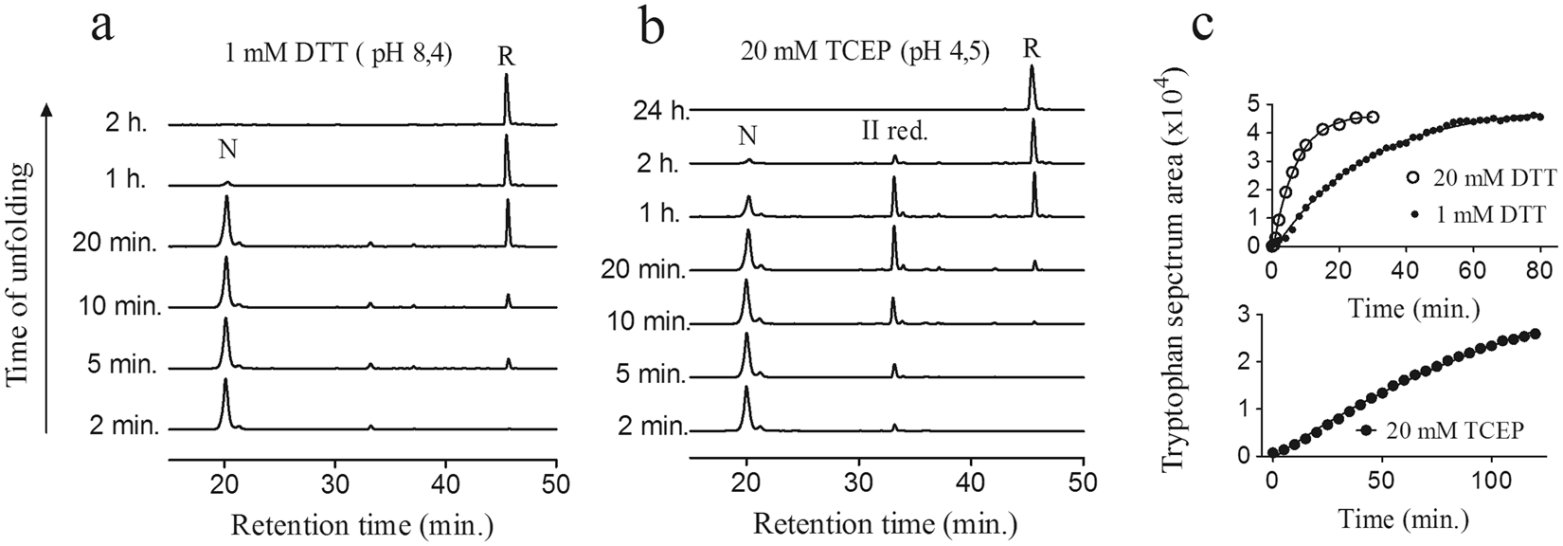
Reductive unfolding of native NvCI. Native NvCI was treated with: (**a**) 1 mM DTT in Tris-HCl (pH 8.4) and (**b**) 20 mM TCEP in sodium acetate (pH 4.5). Time course intermediates were trapped by acidification and analyzed by RP-HPLC. N and R stand for native and reduced NvCI; II for an intermediate that accumulates upon reduction with 20 mM TCEP. (**c**) Reductive unfolding of native NvCI followed by changes in tryptophan fluorescence spectra, after the addition of reducing agents. Native NvCI was reduced with TCEP (20 mM) in sodium acetate pH 4.5 (graph below) and DTT (1.0 and 20 mM) in Tris.Cl pH 8, 4 (graph above). Tryptophan fluorescence spectra were measured over time and the spectrum area was plotted as a function of time.

The reductive unfolding of native NvCI was studied by using both dithiothreitol (DTT) at pH 8.4 and Tris(2-carboxyethyl)phosphine hydrochloride (TCEP) at pH 4.5, as reducing agents. The intermediates that accumulated along the unfolding reaction were trapped in a time-course manner by acidification and analyzed by RP-HPLC. The addition of 1 mM DTT at pH 8.4 resulted in the reduction of the 3S-S native form in one hour (Fig. 4a), while incubation with 20 mM DTT resulted in complete protein reduction in less than 5 minutes (data not shown). Along the process, two minor fractions with HPLC elution times equivalent to that of intermediates I and II were detected. However, the extent of accumulation of these partially reduced species was much lower than that observed in oxidative folding, thus the unfolding reaction followed an apparent all-or-none mechanism^37^.

The use of TCEP at pH 4.5 as a reducing agent avoids disulfide bond isomerization during the reduction process, resulting in a much stronger accumulation of intermediate II (Fig. 4b). The disulfide bond content of this species was assessed by RP-HPLC purification, alkylation and further MS analysis and, as expected, it turned to be a 2-disulfide species.

Reductive unfolding was also followed by monitoring the changes in tryptophan intrinsic fluorescence. The presence of DTT led to an increase in fluorescence intensity concomitant with the progression of the unfolding reaction, without a detectable shift in the spectra maximum wavelength (Fig. 4c, top). As expected, the rate of the fluorescence emission increase was dependent on the concentration of DTT and the maximum spectrum area value was achieved at the time the protein becomes fully reduced. The comparison between the HPLC profile and the tryptophan spectrum of the 20-minute TCEP reduced sample gave us information about conformational properties of the intermediate II. The relative abundances of native NvCI, peak II and the reduced protein are 57%, 36% and 7% respectively, as deduced from the HPLC pattern, but the total change in the tryptophan spectrum only represents a 14% at that time point. These results suggest that the tryptophan remains significantly buried in intermediate II (Fig. 4c, bottom).

### Disulfide-pairing analysis of the major folding intermediates

After examination of the oxidative folding and reductive unfolding of NvCI, it was important to determine the disulfide pairings of the two main intermediates (i.e. I and II) that accumulate during the reactions. The intermediates were purified by RP-HPLC and subjected to a sequential alkylation-reduction-alkylation strategy, as described in methods. The protein reacted with a first alkylating reagent that can only label the available free cysteines and then the sample was reduced and alkylated with a second alkylating reagent, which labelled the cysteines that were initially protected owing to their participation in disulfide bonds. The double derivatized intermediates were subsequently digested with trypsin and subjected to MALDI-TOF and TOF-TOF analysis.

**Figure 5:**
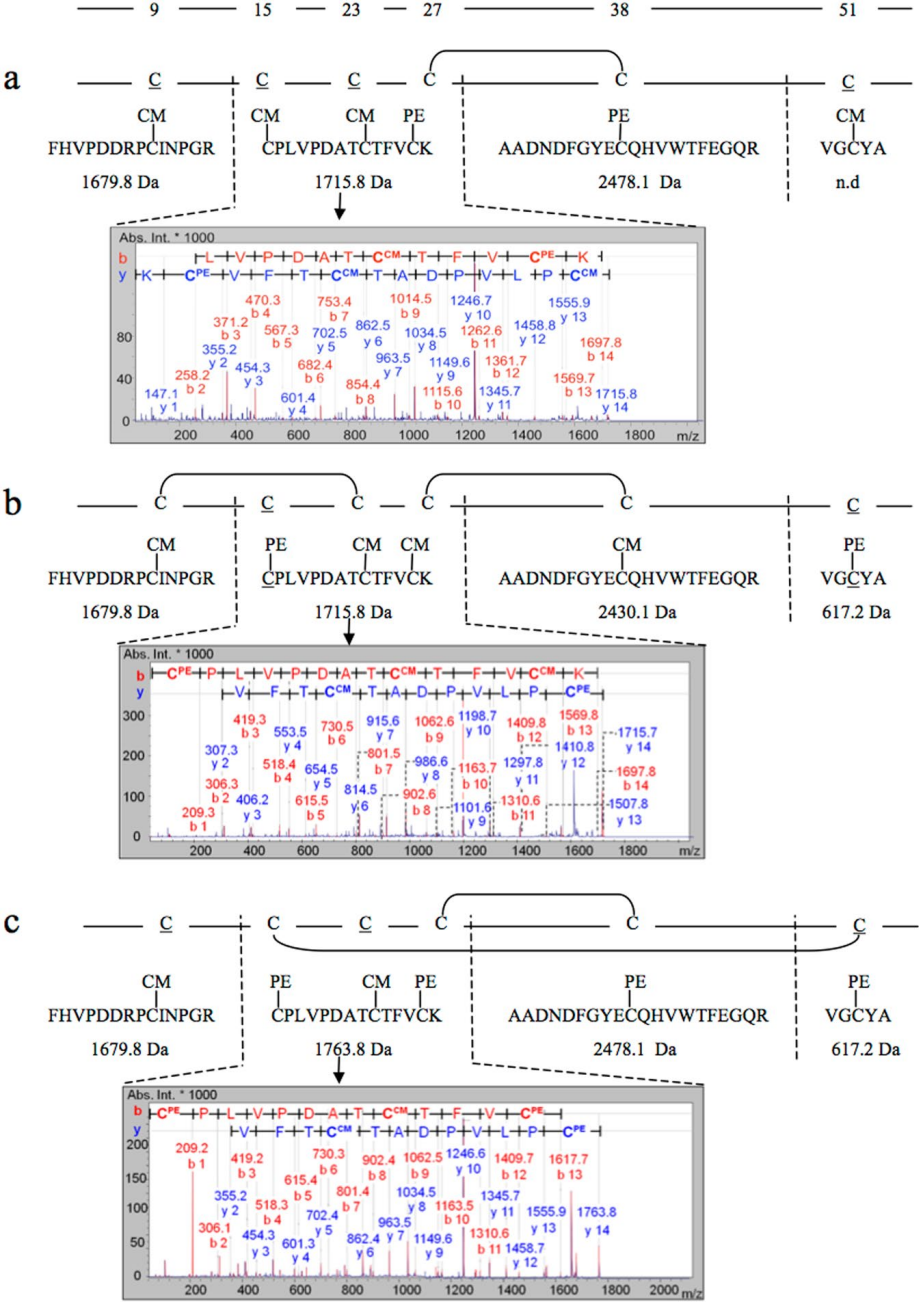
Disulfide-pairing determination of the major oxidative folding intermediates of NvCI. CM stands for carboximethyl cysteine (alkylated with IAA) and PE stands for pyridylethyl cysteine (alkylated with VP). The cysteine positions are indicated above the graph. (**a**) Characterization of fraction I: The purified fraction I was derivatized with IAA, reduced with DTT and further derivatized with VP prior trypsin digestion. The molecular masses determined by MALDI-TOF-MS for the derivatized tryptic peptides are shown. The 1715.8 Da derivatized peptide was sequenced by MALDI-TOF-MS^2^ and the spectrum characterized by β and γ ion series with the assigned sequence is shown. (**b**) Fraction IIb characterization: The purified IIab fraction was derivatized with VP and purified by RP-HPLC (see Fig. 5c, II b-PE). Then it was reduced with DTT, derivatized with IAA and subjected to trypsin digestion and MALDI-TOF analysis. The 1715.8 Da fragment was sequenced by MALDI-TOF-MS^2^ and the spectrum with the assigned sequence is shown. (**c**) Fraction IIa characterization: The purified IIab fraction was derivatized with IAA and purified by RP-HPLC (see Fig. 5, II a-CM). Then it was reduced with DTT, derivatized with VP and subjected to trypsin digestion and MALDI-TOF analysis. The 1763.8 Da fragment was sequenced by MALDI-TOF-MS^2^ and the assigned sequence is shown.

The results obtained for the sequential alkylation strategy of peak I (IAA-DTT-VP) are shown in Fig. 5a. The 2478.1 Da peak corresponded to a peptide with a single cysteine residue (Cys38) that was derivatized with VP, indicating that Cys38 participated in a disulfide bond. The 1715.8 Da peak correlated with a tryptic peptide containing 3 cysteines, two of them derivatized with IAM and one with VP. MALDI-TOF-MS^2^ sequencing of the peptide allowed the assignation of the pyridylethyl modification to cysteine 27 and with this result, it could be inferred that intermediate I contained a single native disulfide bond corresponding to that linking Cys27-Cys38 (Fig. 5a).

**Figure 6:**
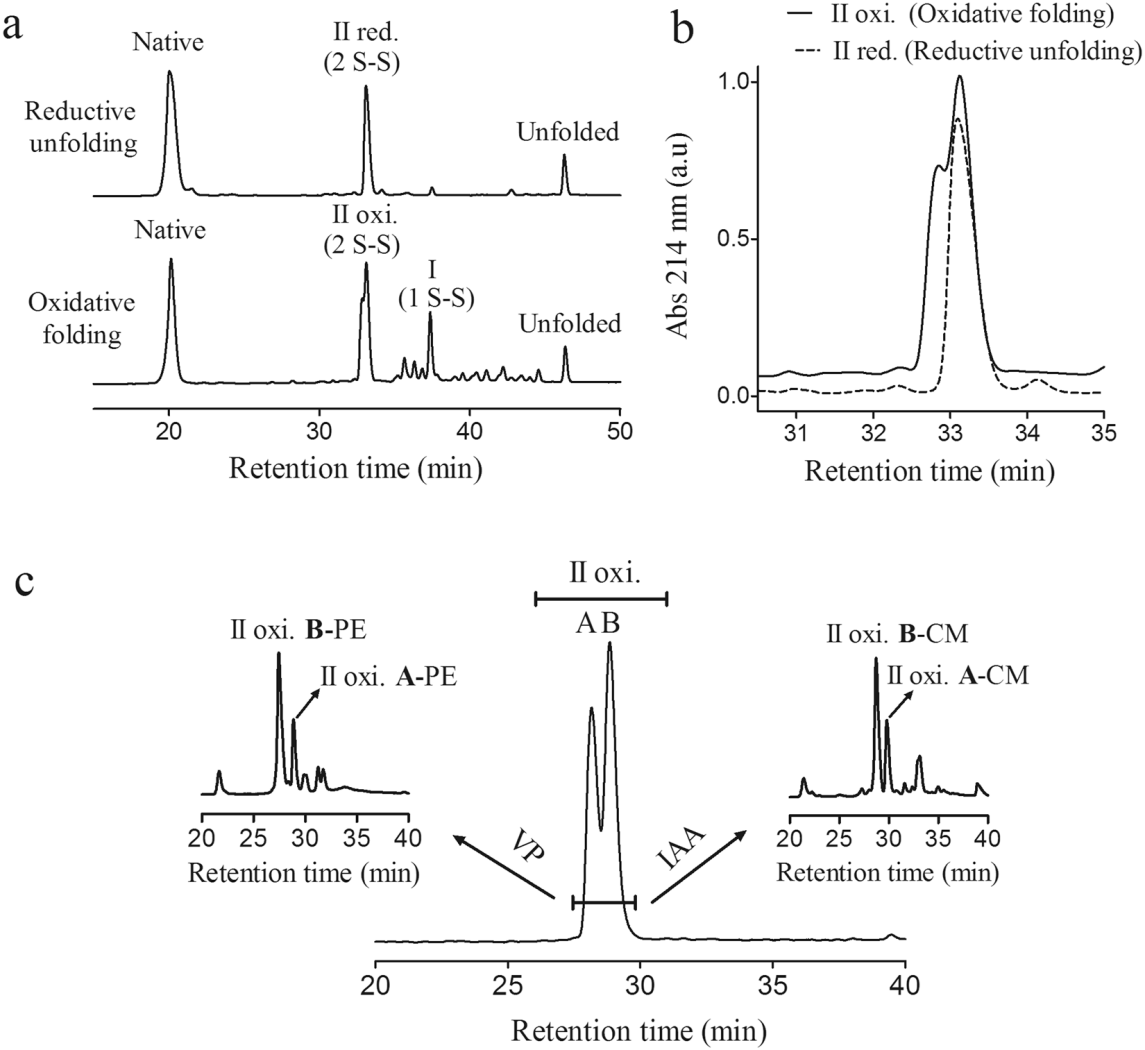
Derivatization and HPLC isolation of two 2 S-S folding intermediates of NvCI. (**a**) Comparison of RP-HPLC profiles of oxidative folding and reductive unfolding. Fraction II of oxidative folding (IIab) and fraction II of reductive unfolding (IIb) elute at the same retention time. The disulfide bond content of all folding intermediates are indicated. (**b**) Superimposition of oxidative folding and reductive unfolding RP-HPLC chromatograms, showing the elution position and the heterogeneity of fraction IIab. The RP-HPLC was performed using a C4 column as described in methods. (**c**) The fraction corresponding to IIab, composed at least by two species (a and b), was purified by RP-HPLC in a C8 column, freeze-dried and derivatized either with VP or IAA. Two major species of each derivatized IIab fraction were further purified by RP-HPLC (left and right insets). An inversion of the order of peaks elution was observed after derivatization. PE stands for pyridylethyl species (akylated with VP) and CM stands for carboximethyl species (alkylated with IAA).

A detailed comparison of the chromatographic profiles of oxidative folding and reductive unfolding of the acid trapped intermediates revealed that the reductive unfolding peak II (designated IIb from now on) was apparently homogeneous, whereas the oxidative folding peak II (designated IIab from now on) was heterogeneous, containing at least two different 2-disulfide species (Fig. 6a and b). Considering the heterogeneity of fraction IIab coming from oxidative folding, it was essential to separate these species for the disulfide pairing analysis. Therefore, the whole fraction IIab was purified by RP-HPLC, subjected to a first alkylation step (VP or IAA) and the resulting species were repurified by RP-HPLC (Fig. 6c). The two main derivatized fractions (a and b) were freeze-dried, reduced with DTT, subjected to a second alkylation step (IAA or VP), digested with trypsin and analyzed by MS. In Fig. 5b, is shown the disulfide pairing characterization of the purified II-b-PE fraction. Starting with a purified intermediate, in which the free cysteine residues had been blocked previously with VP, the disulfide bonds were reduced by DTT and the resultant thiols alkylated with IAA and subjected to trypsin digestion. The 1679.8 Da and the 2430.1 Da peaks corresponded to peptides containing 1 cysteine derivatized with IAM, indicating that their cysteine residues (Cys9 and Cys38) originally participated in disulfide bonds. On the other hand, the 617.2 Da peak matched with a peptide with a single cysteine derivatized with VP (Cys51), implying that Cys51 was free in intermediate IIb. The 1715.8 Da peak, corresponded to a peptide with 3 cysteines, two of them modified with IAM and one with VP. The MS/MS analysis led to the assignation of the cysteine modifications, concluding that Cys15 was the one derivatized with VP and thus, the one that was free in the intermediate, while Cys23 and Cys27 were modified with IAM and thus participated in the disulfide bonding of the IIb form. The same MALDI-TOF-MS and MS^2^ results depicted in Fig. 5b, could have been observed if the intermediate II-b-PE had harboured 2 non-native disulfides. We excluded this possibility when we analyzed by the same strategy, the purified homogeneous fraction IIb obtained from reductive unfolding experiments with 20 mM TCEP at pH 4.5. The fraction IIb elutes in the same position than IIab (Fig. 6b). We knew beforehand that this species could only contain two native disulfide bonds and we obtained exactly the same results as described above. Therefore, the IIb species comprises two native disulfide bonds: Cys9-Cys23 and Cys27-Cys38, and will be designated henceforth des(15–51).

The disulfide pairing characterization of the purified II-a-CM fraction is shown in Fig. 5c. The detection of the 1679.8 Da peak indicated that Cys9 was free, while the 2478.1 and 612.7 Da peaks revealed that Cys38 and Cys51 were forming part of disulfide bonds. The MS/MS sequence analysis of the 1763.8 Da fragment, showed that Cys23 was initially a free cysteine, while Cys15 and Cys27 were disulfide bridged. Thus, the remaining 2-disulfide species of fraction IIab contained the Cys15-Cys51 and Cys27-Cys38 native disulphide bonds, designated from now on as des(9–23).

Integration of the IIab derivatives HPLC peak areas (Fig. 6c, left and right insets) allowed us to estimate that des(15–51) (fraction II-b) accounted for 60–70% while des(9–23) (fraction II-a) corresponded to 30–40% of the native 2-disulfide oxidative folding intermediates. Both intermediates share the native Cys27-Cys38 disulfide present in intermediate I, and no prevalent intermediate without this covalent link was detected.

### Stop-go folding of the major intermediates of NvCI

**Figure 7:**
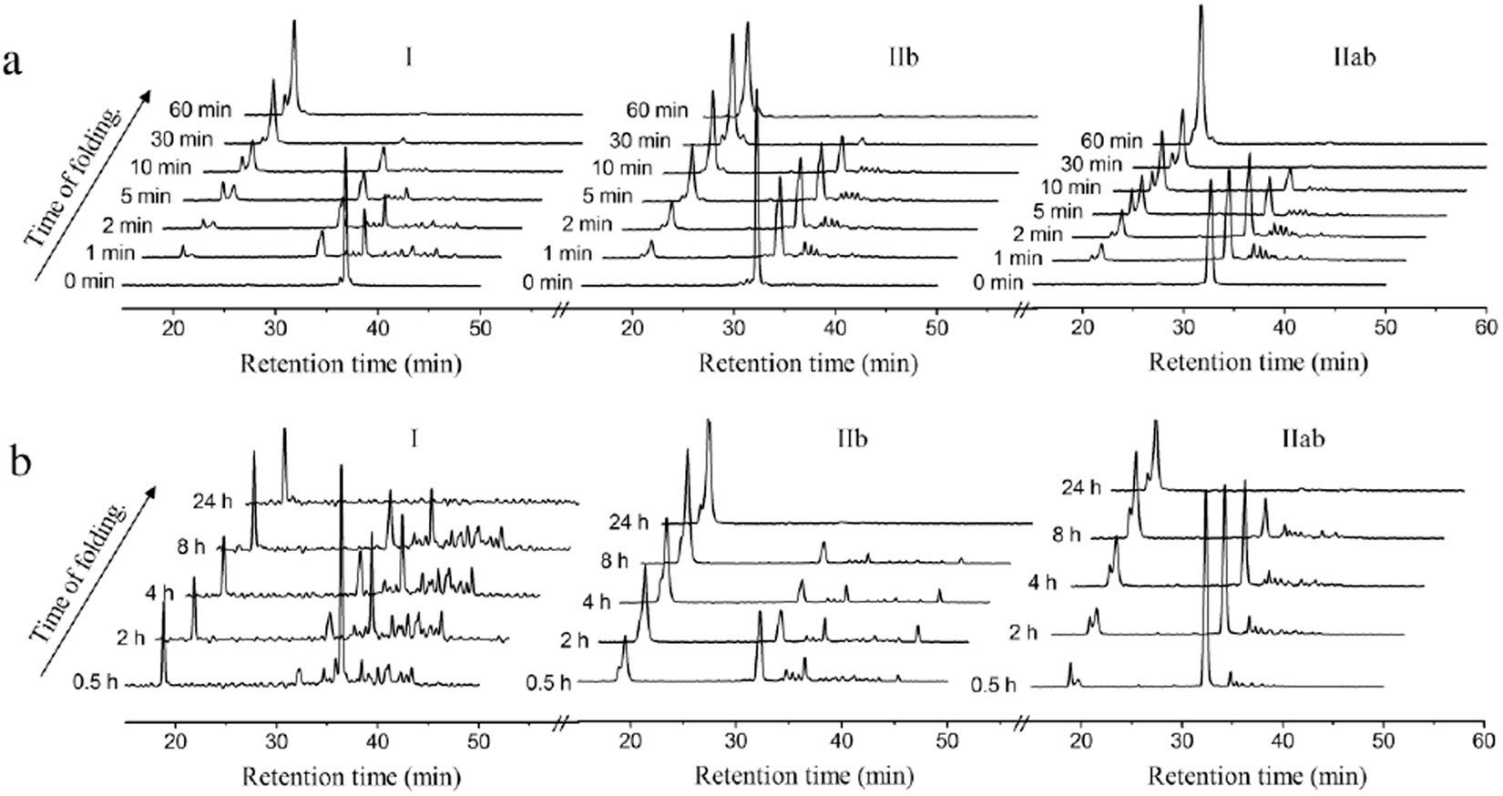
Stop/go folding of the major folding/unfolding intermediates of NvCI. Acid-trapped intermediates (I, IIb and IIab) were purified by RP-HPLC, freeze-dried, and dissolved in Tris-HCl (pH 8.4) to reinitiate the folding reaction either in the absence (**b**) or presence (**a**) of 1 mM GSSG. Folding intermediates were subsequently trapped with acid at different time points and analyzed by RP-HPLC. The chromatograms at 0 minutes (shown in a) were obtained after resuspending the lyophilized intermediates in TFA 1% as a control of purity and homogeneity of each species.

To further assess the kinetic roles of *NvCI* folding/unfolding intermediates, we performed stop-go experiments on the purified species. Fraction I (Cys27-Cys38), IIb (des(15–51) and IIab (comprises des(15–51) and des(9–23)) were freeze-dried and allowed to resume its folding at pH 8.4 in the absence and presence of GSSG. For all tested intermediates, GSSG strongly accelerated the conversion to the native protein, which was almost completed 30 min after resuming folding (Fig. 7a). The stop-go folding profiles of intermediate I, showed that it rapidly reached an equilibrium with fraction IIab and other minor 1 and 2-disulfide isomers, equivalent to the ones observed in NvCI oxidative folding (see Fig. 2a).

The overall refolding profiles of intermediates IIb and IIab in the presence of GSSG were similar. The purified des(15–51) intermediate (IIb) only requires the formation of the Cys15-Cys51 disulfide to yield native NvCI. In case it was the productive species, in the presence of GSSG it would form a mixed disulfide with glutathione and subsequently, a thiol disulfide exchange with the remaining cysteine thiol to form the last native disulfide bridge and render native NvCI. However, after 1 min of refolding, peak broadening was observed, obtaining a similar peak profile to that of fraction IIab at the same time point, which indicates that des(15–51) was in rapid equilibrium with des(9–23). Moreover, the des(15–51) species was also in equilibrium with three minor 2 disulfide isomers, as characterized before (see Fig. 6a, peaks with retention time from 35.5 to 37 min), consistent with the intramolecular rearrangement of des(15–51) to des(9–23) likely involving a reshuffling reaction in which minor 2-species can be populated.

The stop-go folding reaction at pH 8.4 in the absence of GSSG was slower for all NvCI folding intermediates (Fig. 7b). Interestingly, during the refolding of des(15–51) the population of 2 and 1 disulfide native and non-native intermediates as well as of the reduced protein was observed. Therefore, in the absence of an oxidizing agent that accelerates covalent crosslinking, des(15–51) enters in equilibrium with all the species that populate the oxidative folding of NvCI. In contrast, the refolding of fraction IIab in the same conditions neither showed an interconversion with 1-disulfide species, nor with the fully reduced protein.

### Conformational Stability of NvCI

**Figure 8:**
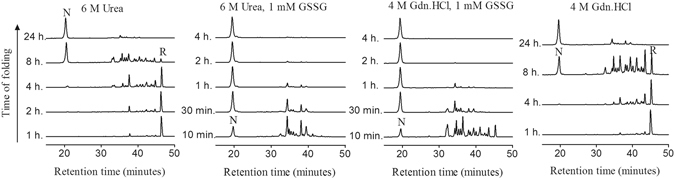
Oxidative folding of NvCI in the presence of high denaturing conditions. The fully reduced/unfolded protein was allowed to refold in Tris-HCl buffer (pH 8.4) in the presence of: 6.0 M urea; 1 mM GSSG in 6.0 M urea; 4.0 M Gdn.HCl and 1 mM GSSG in 4.0 M Gdn.HCl. Intermediates were acid-trapped at the noted times and analyzed by RP-HPLC. The elution positions of native (N) and reduced (R) NvCI are indicated.

The oxidative folding reaction was performed in the presence of 6.0 M urea in 0.1 M Tris-HCl (pH 8.4) and the intermediates were acid-trapped in a time course manner and examined by RP-HPLC. As shown in Fig. 8, in these strongly denaturing conditions, the oxidative folding reaction proceeded without loss of native protein recovery, being almost complete after 24 h of incubation. In comparison with the folding reaction without denaturant (Fig. 2a), the presence of 6.0 M urea decreased the accumulation of native 1-disulfide and 2-disulfide intermediates, while other intermediates, probably comprising non-native 1S-S and 2S-S species populated the reaction. In the presence of 6.0 M urea, the addition of 1 mM GSSG to the folding reaction drastically accelerated native protein recovery that was almost complete after 1 h, a time equivalent to the one needed under non-denaturing conditions (Fig. 2a). NvCI was able to attain its native state even in the presence of 4.0 M guanidine hydrochloride (Gdn.HCl) (Fig. 8), with almost a complete conversion after 24 h of refolding in Tris-HCl pH 8.4. Again, the identity of the intermediates populated along the reaction changed relative to those observed under non-denaturing conditions. As in the case of urea, the presence of 1 mM GSSG greatly accelerated the folding reaction, which proceeded in a timeframe equivalent to this observed under native conditions.

**Figure 9:**
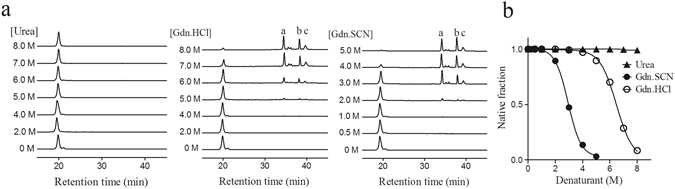
Disulfide scrambling of NvCI under different concentrations of denaturants. (**a**) The native form of NvCI was denatured in Tris-HCl buffer (pH 8.4) containing 0.25 mM 2-mercaptoethanol as thiol initiator and the indicated concentration of denaturants at 20 °C for 20 h. The denatured samples were quenched with 2% TFA and analyzed by RP-HPLC. The fractions a, b and c correspond to non-native (scrambled) isomers of NvCI. (**b**) Native fraction as a function of denaturant concentration. Denaturation of native NvCI is defined by the conversion of the native structure into scrambled isomers. The denaturants are Gdn.SCN (⚫), Gdn.HCl (○) and urea (▲).

Next, we proceeded to analyze the conformational stability of NvCI using the technique of disulfide scrambling. It has been well established that when disulfide-containing proteins are treated with denaturants in the presence of a thiol initiator, their unfolding results in reshuffling of their native disulfide bonds, leading to the formation of disulfide-scrambled species^38, 39^. On this basis, native NvCI was incubated in the presence of 0.25 mM 2-mercaptoethanol as a thiol initiator and increasing concentrations of urea, Gdn.HCl or guanidine thiocyanate (Gdn.SCN) at pH 8.4. The protein mixtures were allowed to reach equilibrium for 20 h, trapped by acidification and analyzed by RP-HPLC (Fig. 9).

The extent of native NvCI unfolding was clearly dependent upon the strength of the denaturant, as previously described for other protein models^38, 40^. Urea was unable to denature native NvCI, and even in the presence of 8.0 M urea no scrambled isomers were detected (Fig. 9a). On the other hand, both Gdn.SCN and Gdn.HCl had a strong effect on NvCI disulfide scrambling promoting the reshuffling of native NvCI into scrambled isomers. The midpoint denaturant concentrations for Gdn.SCN and Gdn.HCl were 3.0 M and 6.5 M, respectively (Fig. 9b). The native protein was completely denaturated at 5.0 M Gdn.SCN and three scrambled isomers, which account for 95% of total denatured NvCI could be distinguished (Peaks a, b and c. Fig. 9a). The same isomers could be observed using Gdn.HCl as denaturant. Peaks a, b and c were purified by RP-HPLC, freeze-dried, reacted either with VP or IAA and further analyzed by MALDI-TOF-MS to confirm the disulfide bond content of the scrambled isomers. As expected, all the scrambled species contained three disulfide bonds and none of them reacted with the alkylating reagents. The high retention of these species in the RP-HPLC profile relative to native NvCI indicate, that under chromatographic conditions, they are significantly less compact.

### Functional and conformational characterization of des(15–51) NvCI

Fraction IIb (des(15–51)) together with the native NvCI protein, were purified by RP-HPLC from the acid-trapped 20 min sample of reductive unfolding with 20 mM TCEP at pH 4.5. The purified intermediate as well as the native protein were quantified by absorbance at 280 nm and freeze-dried. Samples were resuspended at 1 mg/ml in H_2_O/D_2_O (9:1 ratio, pH 2.0) and subjected to monodimensional NMR. The NMR spectrum of des(15–51) displayed a wide signal dispersion of resonances at both low (amide and aromatic region) and high (methyl region) fields, with good peak sharpness, characteristic of a compact molecule, despite the signal dispersion was somehow smaller than that exhibited by the native protein (Supplementary Fig. S2). The comparison of the one-dimensional NMR spectra of NvCI and des(15–51) suggests that the intermediate retains a significant amount of native structure, consistent with its partial resistance to reduction and the quenching of the Trp residue, but is likely more disordered and/or flexible than the native inhibitor.

NvCI is a novel tight-binding, competitive inhibitor of MCPs, which displays the strongest inhibitory constants described (in the picomolar range) against most CPA-type forms^29^. The lower *Ki* values observed for NvCI compared to other MCP inhibitors were previously attributed to both the primary and secondary interaction regions, which create an extended interface with the carboxypeptidase enzyme that minimizes the product release of the catalytic reaction^29^. The primary contact region of the inhibitor corresponds to the C-terminal tail (Tyr52 and Ala53) together with the main chain of Cys51, whereas the secondary contact region is very extended and coincides with the exoface of the NvCI β-sheet.

Inhibition constants for the complexes of native NvCI and the des(15–51) intermediate with different carboxypeptidases were determined (Table 1). The inhibitory activities of the intermediate des(15–51) against human CPB1 and porcine CPB1 were one order of magnitude lower than that of NvCI, but the intermediate still acts as a tight-binding inhibitor for B type carboxypeptidases, with *Ki* in the nanomolar range. Native NvCI showed inhibition constants in the picomolar range for human CPA1 and bovine CPA1, in agreement with our previous data^29^. The inhibitory capability of the intermediate des(15–51) for both type A tested carboxypeptidases was three orders of magnitude lower than the native form, but again it displayed *Ki* in the nanomolar range, showing that it behaves as a tight-binding inhibitor for these enzymes. This is relevant because the Cys15-Cys51 disulfide bond was supposed to immobilize the C-tail of NvCI as a requirement for a proper inhibition. Interestingly, the side chain of Glu-163 in hCPA4 forms favorable hydrogen bond contacts with the amino groups of Cys-51 and Tyr-52 of NvCI^29^.

**Table 1:** Summary of *K*_*i*_ values of native and des(15–51) intermediate against various carboxypeptidases.

Carboxypeptidase type	NvCI	des(15–51)
	*Ki* (nM)	
hCPA1	0.00118 ± 0.00039	1.00 ± 0.08
bCPA1	0.00491 ± 0.00064	2.01 ± 0.14
hCPB1	0.62 ± 0.04	7.85 ± 0.55
pCPB1	0.53 ± 0.04	4.90 ± 0.28

Data are means (n = 3) ± S.D. Inhibition assays were carried out at 37 °C.

## Discussion

Different models have been proposed to explain the cooperativity of protein folding transitions. Seminal oxidative folding studies by the laboratories of Creighton, Kim, Scheraga, and Chang using BPTI, RNase A and hirudin as model proteins contributed significantly to the experimental validation of these theoretical models^3, 10, 25, 41^. The framework model and the hydrophobic collapse model represent two extreme canonical and generic descriptions of the protein folding process. The BPTI folding is in line with the framework model^42, 43^, which stresses the importance of local interactions in reducing conformational search and in guiding efficient protein folding through the hierarchic condensation of native-like elements. In contrast, the hirudin folding is consistent with the hydrophobic collapse model^44^, which depicts protein folding as an initial stage of rapid hydrophobic collapse followed by searching of conformations in which specific interactions refine the structure rather than dominate the folding code^22, 45^. It is still unclear which features of the primary structure of a small, disulfide-rich protein, influence its type of folding, since proteins sharing the same conformation and disulfide connectivity fold through different pathways and, conversely, proteins with different structural features turn to share the same type of folding reaction^46^. This fact highlights the need for new and detailed studies to gain insights into the molecular rules that govern the folding process.

In the current work we have comprehensively analyzed the pathways of oxidative folding and reductive unfolding of NvCI. Although 75 different disulfide-bonded intermediates are theoretically possible, only a few species populate significantly the oxidative folding reaction of this inhibitor. Importantly, all major intermediates contain one or two native disulfide bonds, suggesting a strong bias toward the establishment of native-like interactions during folding under close to physiological conditions. Therefore, for NvCI, folding appears to proceed through the sampling of a reduced number of specific metastable states rather than through a large conformational search among species of similar free energy. CD and Trp fluorescence data indicates that this progressive acquisition of native disulfide bonds is concomitant with the formation of native secondary and tertiary NvCI structure. The presence of a discrete number of natively bonded intermediates, together with the lack of significant scrambled isomers, would account for the high efficiency of NvCI folding, when compared with proteins of similar size and disulfide content^47^. It is worth to clarify, however, that despite its relative efficiency, the *in vitro* folding of NvCI takes hours to complete. It is expected that during catalysed cotranslational oxidative folding in the endoplasmic reticulum the reaction would be completed in significantly shorter times; despite some evidences indicate that even in these favourable conditions disulfide-containing proteins fold much slower than proteins devoid of this covalent link^48^.

**Figure 10:**
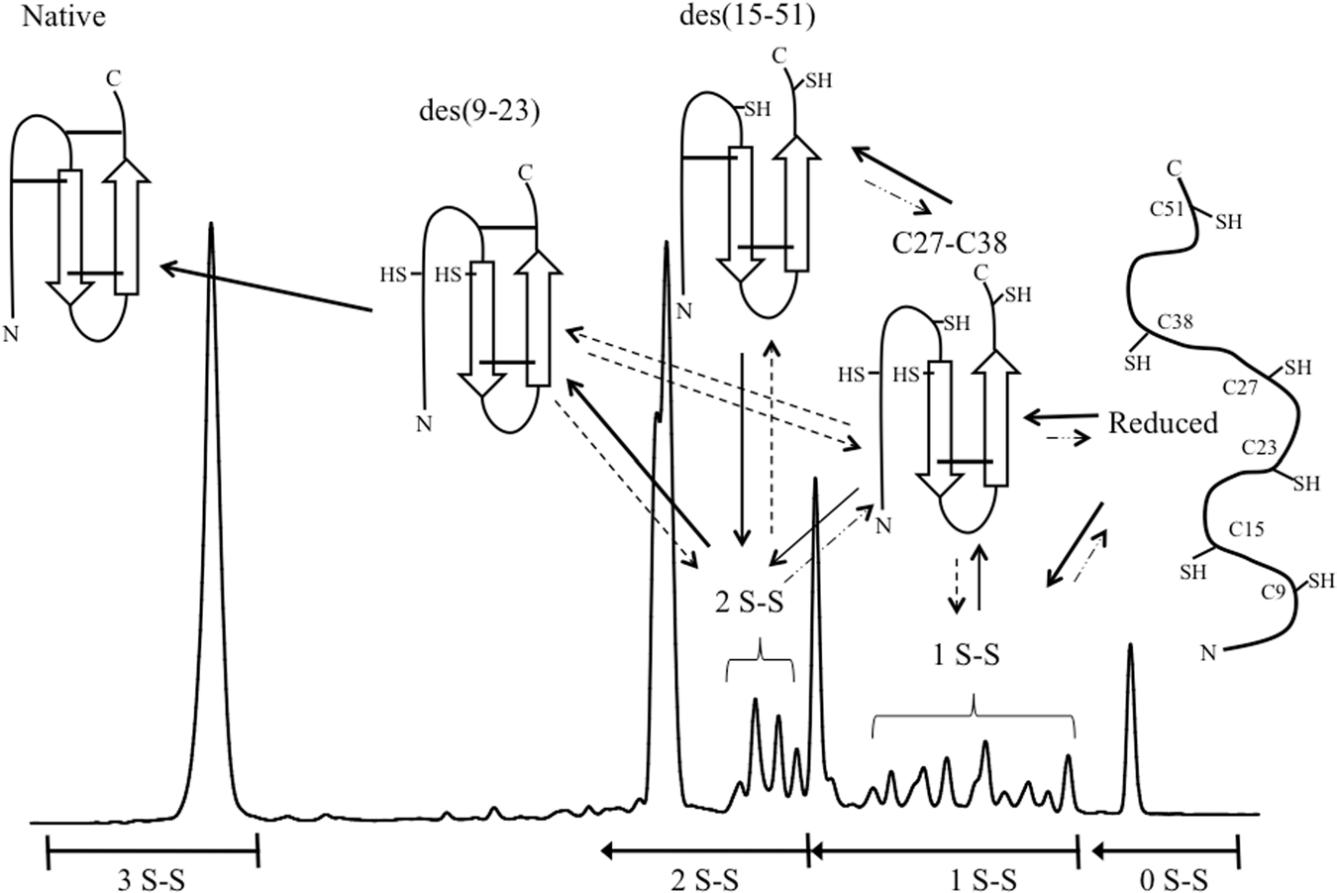
Schematic representation of the disulfide oxidative folding pathway of NvCI. The polypeptide backbone and the β-sheets of the protein are depicted by lines and arrows, respectively. The positions of the six cysteine residues are indicated (SH: 9, 15, 23, 27, 38 and 51) and the disulfide bonds are depicted as solid lines. The RP-HPLC chromatogram and the major disulfide intermediates are depicted and the disulfide bond content of all the RP-HPLC fractions are indicated below the graph. The solid lines represent the productive disulfide folding pathway proposed.

The predominant folding pathway followed by NvCI at pH 8.4 is summarized in Fig. 10. Initially, reduced NvCI is oxidized to a mixture of one-disulfide species, with a high prevalence of the Cys27-Cys38 form that comprises the shortest loop closed by native disulfides. Thus, the configurational entropic contribution of this linkage to the stability of one-disulfide intermediates is the lowest among those provided by native disulfide bonds. The abundance of this intermediate cannot be explained simply on the basis of the sequential vicinity of Cys, because in this case one-disulfide intermediates containing the non-native Cys23-Cys27, Cys9-Cys15 or Cys15-Cys23 bonds would be more prevalent and they could not be detected. Therefore, the population of the Cys27-Cys38 species likely responds to the establishment of preferential noncovalent interactions between residues surrounding these specific Cys residues. This disulfide bond links the C-terminus of the β-strand 1 to the N-terminus of the adjacent β-strand 2 in the antiparallel β-sheet of NvCI, the only regular secondary structure in this small protein. Prediction of the secondary structure propensities in NvCI sequence using GOR V^49^ indicates an intrinsic β-sheet propensity for the segments 24–28 and 36–45, whereas PSIPRED^50^ predicts the stretches 24–29 and 37–45 as β-strands, both predictions overlapping with the two β-strands in the NvCI crystal structure, which comprise residues 22–29 and 36–44, respectively. In agreement with the crystal structure, none of the two algorithms predict secondary structure propensity in other regions of the NvCI sequence. Both Cys27 and Cys38 are inside the predicted β-strands, suggesting that a significant secondary structure bias in these stretches is responsible for the initial formation of the disulfide bond in the Cys27-Cys38 species. Subsequently, the covalent crosslink would contribute to stabilize the β-sheet.

Three main factors influence the reactivity of a disulfide bond, i.e, its electrostatic environment, the geometry of the bond and its burial. We minimized the isolated inhibitor structure using the Fold-X force field^51^ to analyze the potential contribution of these factors to the reactivity of the Cys27-Cys38 disulfide. No charged residues exist close to this covalent link that could affect its electrostatic environment, but the disulfide strain energy in the native state for this bond is very high (15.77 kJ/mol), suggesting that it would be reactive if accessible to solvent. This indicates that it should be at least partially buried to allow the accumulation of intermediate I as the major species in the 1S-S ensemble. The formation of a native-like β-sheet in the intermediate might account for this effect, since, in the native state, it completely buries Cys27, with less than 2% of its side chain exposed to solvent. Nevertheless, stop-go experiments on the isolated intermediate indicate that, despite its prevalence, the Cys27-Cys38 disufide bond is not completely secure and reshuffling reactions allow the formation of other 1S-S species at the beginning of the refolding reaction, especially in the absence of GSSG. Indeed, intermediate I does not accumulate during reductive unfolding reactions, even at acidic pH, which indicates that, in the absence of the other two native disulfides, at least one of its two Cys becomes accessible to the reducing agent. Thus, disulfide stapling of the β-sheet does not guaranties, *per se*, its stability.

In the next step of the NvCI oxidative folding, one-disulfide intermediates oxidize a second pair of cysteines to form two-disulfide intermediates that accumulate mainly as des(9–23) (intermediate IIa) and des(15–51) (intermediate IIb), both of them containing two native disulfide bonds and sharing the Cys27–Cys38 crosslinking; thus suggesting that they evolve directly from oxidation of intermediate I. Despite RP-HPLC analysis indicate that these two 2S-S intermediates should display very similar compactness, des(15–51) is the only intermediate that populates the reductive unfolding reaction at acidic pH in the presence of TCEP, where reshuffling is impeded; thus indicating that the 9–23 disulfide bond is less reactive in the native inhibitor than the 15–51 one. An inspection of the NvCI structure suggests that both disulfide bonds are protected from the solvent to approximately the same extent, with Cys9 and Cys15 deeply buried in the structure. However, in the crystal structure of the inhibitor/enzyme complex, Cys15-Cys51 disulfide is covered by the inhibitor C-tail only because it is docked and fixed in the active site of the carboxypeptidase moiety. We used the CABS-FLEX^52^ algorithm to simulate the structural flexibility of non-bound NvCI and the generated structural models strongly suggest that the C-tail would be flexible in solution and not expected to establish significant contacts with the rest of the domain (Supplementary Fig. S3), as described for most MCPs inhibitors, exposing at least partially the Cys15-Cys51 disulfide bond. From an entropic point of view, des(9–23) would be expected to be less destabilized, relative to the native state, that des(15–51) since the Cys15-Cys51 disulfide bond closes by far the longest loop in NvCI. The log n rule of Darby and Creighton^53^, predicts a destabilization of 2.9 and 3.8 kcal·mol^−1^ for des(9–23) and des(15–51), respectively; thus supporting that local disulfide protection, in at least a partially buried context and not the overall conformational stability of the intermediate would account for the higher resistance to reduction of the des(15–51) species. This is consistent with des(15–51) retaining a compact native-like conformation according to both NMR and carboxypeptidase inhibition assays.

Unexpectedly, stop/go experiments indicate that des(15–51) is not the productive 2S-S species, since NvCI native structure cannot be simply acquired by direct oxidation of the free Cys15 and Cys51 thiol groups. Indeed, isolated des(15–51) loss one or even two of its native disulfides before the reaction can proceed towards the formation of IIab and, latter on, towards the native ensemble, indicating that its disulfide bonds are available for intramolecular attack by their own thiols and, thus, that it is a disulfide-insecure intermediate. In the absence of the Cys15-Cys51 entropic restriction, the segment Gln39-Ala53 lacks any covalent connectivity with the rest of the molecule and would likely experiment conformational fluctuations that could conduct the free Cys51 to transiently reside close to preformed disulfide bonds, promoting reshuffling reactions. This agrees with the observation that, when reductive unfolding is performed at pH 8.4, where reshuffling is permitted, this intermediate does not accumulate anymore. This will explain why, during the folding reaction, the conversion of 2S-S species towards the native state is accelerated in the presence 2-mercaptoethanol (Fig. 2), since it favours reshuffling and also why when oxidation is favoured in the presence of GSSG, natively cross-linked 2S-S species accumulate significantly before they can be converted into native NvCI. The formation of des(9–23) from des(15–51) involves the population of detectable less compact non-native 2S-S intermediates, even in the presence of GSSG, but these species do not accumulate significantly. It is likely that des(9–23) would act as the productive 2S-S species, which would be consistent with the non productive des(15–51) intermediate being the more abundant species in the IIab fraction. However, the impossibility to purify des(9–23) in an unmodified form to perform stop/go analysis does not allow to fully discard a potential involvement of non-native 2S-S species in the formation of functional NvCI.

Although under physiological conditions the folding reaction of NvCI is under kinetic control, the formation of metastable natively-linked 2 disulfide intermediates is not a requirement for the attainment of the functional structure of this small protein. As evidenced by the fact the protein folds efficiently to the native state even in the presence of 6.0 M urea or 4.0 M Gdn.HCl, where no preferential intermediate accumulates but rather a heterogeneous mixtures of conformers populates the reaction. Disulfide scrambling experiments indicate that in this stringent conditions the protein is folded, suggesting that the folding of NvCI can also be under thermodynamic control. In these conditions, it is difficult to postulate that the formation of the small secondary structure in NvCI would funnel the reaction by establishment of native-like preferential interactions and a mechanism of “trial and error” would more likely apply. Despite the presence of multiple intermediates, the formation of NvCI in the presence of denaturants is fast and efficient. This suggests that the lack of specific noncovalent interactions is compensated by the fast formation and rearrangement of disulfide bonds within the different populations of intermediates and likely by the absence of scrambled isomers, which usually delay folding reactions. Indeed, the scrambled isomers formed under disulfide scrambling conditions are significantly more retained in the RP-HPLC column than the native form, indicating that are significantly less compact than the functional form and that in case they would exist their disulfides would be significantly more exposed.

The ability to fold into the native state under strong denaturing conditions is not unique of NvCI and other small disulfide-rich proteins have been shown to attain the native state to some extent in the presence of chaotropic agents. However, the efficiency of NvCI is unprecedented. PCI exhibit an efficiency of 2.5 and <1% in the presence of 8.0 M urea and 5.0 M Gdn.HCl, respectively^40^. For hirudin, 8% of active protein is recovered in 5.0 M Gdn.HCl^54^. In these conditions, the initial intermediates are likely formed stochastically and the initial nonspecific collapse would represent a first rate limiting step for the formation of the first disulfide bonds. The sequence of NvCI is one of the most hydrophobic among small disulfide-rich proteins, with a grand average hydrophobicity score (GRAVY) value of −0.27, whereas PCI and hirudin display GRAVY values of −0.64 and −0.88, respectively. The higher hydrophobicity of NvCI might well account for a more efficient collapse at the early stages of folding, resulting in the fast formation of a mixture of native and non-native disulfides that would subsequently rearrange to attain the thermodynamically more stable native topology. Indeed, the conformational stability of NvCI, as measured by the method of disulfide scrambling, is one of the highest among small proteins with three disulfides (Supplementary Table S1), being comparable to that of BPTI.

The folding reaction of NvCI provides an extreme example of the plasticity of folding pathways, since the functional native state can be attained following different folding routes that hinder on very different folding mechanisms being under kinetic or thermodynamic control depending on the protein microenvironment. It is largely assumed that the same interactions that stabilize the native structure also guide folding. However, certain proteins can arrive to the same folded state following alternative pathways involving a different succession of native-like interactions^55^. The case of NvCI is more dramatic since it can fold both through a succession of native-like contacts or independently of this kind of interactions. Thus for NvCI it is both true that protein conformational folding drives disulfide formation and the other way around. However, it is unlikely that these two alternative pathways could coexist under physiological conditions, since single molecule force spectroscopy studies in the presence of oxidative folding catalysts like PDI and DsbA strongly support conformational folding preceding Cys oxidation^56, 57^.

Overall, in the present work we delineate the determinants of the foldability, stability and activity of NvCI, providing the basis for the further re-design of this tight binding inhibitor.

## Methods

### Protein expression and purification

Recombinant NvCI was overexpressed and secreted into the extracellular medium using the *Pichia Pastoris* heterologous system as previously described^29^. After centrifugation at 6000 g for 15 min the culture supernatant was adjusted to pH 3.2 with HCl in 0.02 M sodium citrate buffer and filtered through a 0,8 µm membrane filter (Millipore). The culture supernatant was then loaded onto an ion-exchange chromatography column (1.6 × 10 cm, STREAMLINE Direct HST, GE Healthcare) connected to an AKTA purifier system (GE Healthcare). The column was equilibrated with 0.1 M sodium citrate (pH 3.2) and elution was performed increasing gradually the pH applying a 20 column volumes lineal gradient, from 0% to 100% 0.1 M sodium phosphate (pH 8.0). The protein sample was then subjected to a size exclusion chromatography in a HiLoad 26/60 superdex 30 prep-grade column equilibrated in PBS buffer. The purity of the isolated inhibitor was determined by Tris-Tricine/SDS-PAGE, RP-HPLC and matrix-assisted laser desorption/ionization time-of-flight (MALDI-TOF) mass spectrometry (MS). The concentration of purified NvCI was determined by measuring the absorbance at 280 nm using a molar extinction coefficient of 8855 M^−1^ cm^−1^.

### Oxidative Folding and Reductive Unfolding Experiments

Purified native NvCI (1 mg/ml) was reduced and unfolded by incubation in 0.1 M Tris-HCl (pH 8.4) containing 6.0 M GdnHCl and 200 mM DTT for 3 h at RT. To initiate refolding, we loaded the fully reduced/unfolded protein onto a HiTrap desalting column (GE Healthcare) connected to an AKTA purifier system that was previously equilibrated with 0.1 M Tris-HCl (pH 8.4). The protein was eluted in 1.2 ml of equilibration buffer (protein concentration ~0.5 mg/ml) and incubated at RT in the absence (Control−) and in the presence of redox agents: 0.25 mM 2-mercaptoethanol (Control+), 0.5 mM GSSG, or 0.5 mM/1.0 mM GSSG/GSH. Some refolding experiments were carried out in the presence of denaturants, in these cases, the eluted protein was incubated in 6.0 M urea or 4.0 M Gdn.HCl, in the presence or absence of 0.5 mM GSSG. The refolding reaction was monitored by removing aliquots of the sample at various time intervals and quenching them with 2% aqueous trifluoroacetic acid (TFA). Acid-trapped intermediates were subsequently analyzed by RP-HPLC using a linear 20–40% gradient of acetonitrile with 0.1% TFA over 50 min in a 4.6-mm C4 column (Phenomenex) at a flow rate of 0.75 ml/min. The reactions were performed in triplicates at 25 °C.

In reductive unfolding experiments, native NvCI (0.5 mg/ml) was incubated at RT in either 0.1 M Tris-HCl (pH 8.4) containing different concentrations of DTT (1.0 and 20 mM) or 0.1 M sodium acetate (pH 4.5), with 20 mM TCEP. To monitor the unfolding reaction, time course aliquots of the samples were trapped with 2% TFA acid and similarly analyzed by RP-HPLC in a C4 column. To monitor the disulfide species along the unfolding process, folding intermediates were trapped in a time-course manner by alkylation with either 0.1 M 4-vynilpyridine (VP) or 0.1 M iodoacetamide (IAA) for 45 min at RT in the dark. The derivatized samples were diluted 1:10 in 1% TFA acid to stop the reaction and analyzed by MALDI-TOF-MS. Each free cysteine that reacts with VP (pyridylethyl (PE) cysteine derivative) or IAA (carbamidomehtyl (CM) cysteine derivative) increases its molecular mass by 105.1 Da or 57.0 Da, respectively. The percentage of disulfide species along the folding process was calculated by integrating the peak areas corresponding to each disulfide species observed in the mass spectrum. The reductive unfolding experiments were performed in duplicates at 25 °C.

### Disulfide-bond content and disulfide pairing analysis of the major intermediates of oxidative folding and reductive unfolding of NvCI

The acid-trapped intermediates were purified by RP-HPLC and freeze-dried. The free cysteines of the purified intermediates were blocked by derivatization with an alkylating reagent. Each sample (20–40 µg) was derivatized with either 0.1 M VP or 0.1 M IAA, in Tris–HCl (pH 8.4) at RT for 45 min in the dark. An aliquot of the samples was analyzed by MALDI-TOF-MS, to characterize the number of disulfide bonds of the folding intermediates. The derivatized samples were then freed form reagents using Zip-Tip C4 pipette tip (Millipore), evaporated, and the disulfide bonds were reduced by resuspending the dried samples in 10 µl of 10 mM DTT in 0.1 M Tris-HCl (pH 8.4) and incubating for 1 h at RT.

The free cysteines, corresponding to those initially involved in disulfide bridges, were then subjected to a second alkylation reaction by adding 10 µl of the alkylating reagent not used in the first reaction and the samples were incubated for 1 h at RT in the dark. Then, the double-derivatized samples were purified by Zip-Tip C4 and air-dried. The dried samples were resuspended in 10 µl of ammonium bicarbonate pH 8.8 containing 0.15 µg of MS grade trypsin (Trypsin Gold, Promega) and were incubated for 16 h at at 37 °C. The resulting proteolytic derivatized peptides were detected by MALDI-TOF-MS. A tryptic peptide of NvCI containing three derivatized cysteines was sequenced by MALDI-TOF-MS^2^ to determine which cysteines were initially free (derivatized with the first alkylating reagent) or disulfide-bridged (derivatized with the second alkylating reagent).

The acid-trapped fraction IIab from oxidative folding was purified by RP-HPLC using a 4.6 mm Nova Pack C8 cartridge (Waters) and a linear gradient from 20–40% of acetonitrile with 0.1% TFA over 50 min at 0.75 ml/min (See Fig. 6c). The purified heterogeneous fraction was freeze-dried and derivatized by resuspending it in 90 µl of 0.1 M Tris-HCl (pH 8.4), containing either 0.1 M VP or 0.1 M IAA, and was incubated at RT for 45 min in the dark. Then, the derivatized products were separated and purified by RP-HPLC using the same C8 column and conditions described before. The purified derivatized fractions were freeze-dried and subjected to reduction with 10 mM DTT, followed by a second alternative alkylation step and further subjected to trypsin digestion as described above. The resulting derivatized digestion peptides were analyzed by MALDI-TOF-MS and sequenced by MS^2^ as described before.

### Stop/Go folding

Acid-trapped intermediates were isolated by RP-HPLC, lyophilized, and allowed to reinitiate folding at 0.3 mg/ml in 0.1 M Tris-HCl (pH 8.4) at RT in the absence and in the presence of 0.5 mM GSSG. To monitor the stop/go reaction progression, time-course aliquots of the samples were trapped with 2% TFA acid and analyzed by RP-HPLC in a C4 column using the same gradient described in oxidative folding. The fraction IIb was purified from the 20 min acid-trapped sample of reductive unfolding with 20 mM TCEP at pH 4.5. Fractions I and IIab were purified from the 10 min acid-trapped sample of oxidative folding in the presence of 0.5 mM GSSG. The stop-go experiments were performed in duplicates at 25 °C.

### Disulfide scrambling

The native protein (0.5 mg/ml) was dissolved in 0.1 M Tris-HCl (pH 8.4) containing 0.25 mM 2-mercaptoethanol and increasing concentrations of denaturants: urea (0–8 M), GdnHCl (0–8 M) or GdnSCN (0–5 M). The samples were incubated for 20 h at RT to reach equilibrium and then quenched with 2% TFA acid and analyzed by RP-HPLC, as detailed in oxidative folding. The disulfide scrambling experiments were performed in duplicates at 25 °C.

### Circular Dichroism, Fluorescence and NMR spectroscopy

For oxidative folding, fully reduced/unfolded NvCI was prepared as described before, allowed to air oxidize for 16 hours and the changes in the secondary and tertiary structure were monitored by far UV-circular dicroism (CD) and tryptophan fluorescence spectroscopy, respectively. The sample for CD spectroscopy was prepared by dissolving the protein to a final concentration of 0.1 mg/ml in 20 mM Tris-HCl (pH 8.4). CD spectra were collected at 20 min intervals in a spectropolarimeter (Jasco J-710) at 25 °C using a cell of 1-mm path length. Samples for fluorescence spectroscopy were measured in the same buffer on a fluorescence spectrophotometer (Jasco FP-8500) at a final protein concentration of 0.05 mg/ml at 25 °C. The spectra were measured every 20 min in the 310–450 nm interval using a 294-nm excitation wavelength (5 nm excitation and emission slits, 0.1 s averaging time).

For reductive unfolding, native NvCI samples were prepared at 0.03 mg/ml in 50 mM Tris-HCl (pH 8.4) or 100 mM sodium acetate (pH 4.5). After measuring the native protein spectrum, the reducing agent was added, without diluting the samples, to a final concentration of 1.0 mM and 20 mM for DTT (pH 8.4) or 20 mM for TCEP (pH 4.5). Tryptophan emission spectra were measured at various time points in the same conditions described for oxidative folding.

One-dimensional ^1^H nuclear magnetic resonance (NMR) spectra were acquired at 25 °C on an Avance 600-MHz NMR spectrometer (Bruker). Samples coming from the RP-HPLC purified intermediate IIb of reductive unfolding (obtained by reduction with 20 mM TCEP at pH 4.5) and native NvCI were prepared by dissolving the lyophilized purified proteins to a final concentration of 1.5 mg/ml in H_2_O/D_2_O (9:1 ratio, v/v) at pH 2.0.

### MALDI-TOF analyses

MALDI-TOF mass spectra were recorded on an UltrafleXtreme mass spectrometer (Bruker Daltonics) and the samples were spotted onto a MALDI-TOF-MS ground steel plate using the dried-droplet method. For peptide analyses the samples were mixed with α-cyano-4-hydroxycinnamic acid (hcca) and acquired in the positive ion reflectron mode, with ion acceleration set to 25 kV. All mass spectra were externally calibrated using a standard peptide mixture (Bruker Daltonics). For protein analyses the samples were mixed with sinapic acid and acquired in the positive ion lineal mode, with ion acceleration set to 25 kV. All mass spectra were externally calibrated using a standard protein mixture ranging from 4 kDa to 20 kDa (Bruker Daltonics). For tandem MS analyses samples were mixed with hcca and acquired in positive LIFT reflectron mode with ion acceleration set to 7.5 kV and LIFT voltage set to 19 kV.

### Determination of MCPs inhibitory activities of native NvCI and des(15–51) intermediate

Bovine carboxypeptidase A (bCPA1) and porcine carboxypeptidase B (pCPB1) were purchased from Sigma. Human carboxypeptidase A1 (hCPA1) and human carboxypeptidase B1 (hCPB1) were produced by recombinant expression as previously described^35, 58^.

The inhibitory activity of native NvCI and des(15–51) folding intermediate were assayed by measuring the inhibition of the hydrolysis of the chromogenic substrate N-(4-methoxyphenylazoformyl)-Phe-OH by CPAs and N-(4-methoxyphenylazoformyl)-Arg-OH (Bachem) by CPBs at 355 nm. The inhibition assays were performed in 20 mM Tris-HCl (pH 7.5), 1% v/v DMSO, 0.05% w/v Brij-35 with 0.5 M NaCl for CPAs or 0.1 M NaCl for CPBs. The assay was performed at 37 °C in a 96-well microplate format with a final volume of 250 µl adding 20 µl of the substrate to start the reaction, at a final concentration of 0.1 mM. The reactions were followed at 30 sec interval for 15 min and measured in terms of initial velocities in a multiplate reader Wallac 1420 VICTOR2 (PerkinElmer). The enzyme concentrations were kept constant (7.5 nM for hCPA1 and 6.0 nM for bCPA1, hCPB1 and pCPB1) and increasing amounts of the inhibitors were added in each case. Inhibition constants (*Ki*) were determined according to the method described for tight-binding inhibitors^59^. The best-fit value of (*Ki*) was performed by adjusting the experimental values to the Morrison equation^60^ using the program GraphPad Prism 5 (GraphPad Software, Inc.) at p < 0.05. Data are means (n = 3) ± S.D. The concentration of the purified solutions of native NvCI and des(15–51) were determined from the absorbance at 280 nm using a molar extinction coefficient of 8855 M^−1^ cm^−1^.

## Electronic supplementary material

Plasticity in the Oxidative Folding Pathway of the High Affinity Nerita Versicolor Carboxipeptidase Inhibitor (NvCI)
