# Supplementary material for: Plasticity in the Oxidative Folding Pathway of the High Affinity Nerita Versicolor Carboxypeptidase Inhibitor (NvCI)
Source: Sci Rep. 2017 Jul 14;7:5457. doi: 10.1038/s41598-017-05657-7 (PMC5511257; doi:10.1038/s41598-017-05657-7)
Supplement: Supplementary file 1 — Plasticity in the Oxidative Folding Pathway of the High Affinity Nerita Versicolor Carboxipeptidase Inhibitor (NvCI) [file 41598_2017_5657_MOESM1_ESM.pdf]

Plasticity in the Oxidative Folding Pathway of theHigh Affinity *Nerita Versicolor* Carboxipeptidase Inhibitor (NvCI)

Sebastián A. Esperante, Giovanni Covaleda, Sebastián A. Trejo, Sílvia Bronsoms, Francesc X. Avilés* and Salvador Ventura*

Proteins	Size of proteins (residues)	GdnSCN (M)	GdnHCl (M)	Urea (M)
NvCI	53	3.0	6.5	>8
BPTI[1]	58	3.25	7.5	>8
PCI[2]	39	0.7	1.45	>8
Hirudin[3]	49	2	5	>8
EGF[4]	53	2.1	3.9	>8
IGF-1[5]	70	1.5	3.2	5.5

Table Supplementary S1. The concentration of denaturant required to denature 50% of the 3S-S proteins determined by the method of disulfide scrambling. The extent of denaturation corresponds to the fraction of native protein converted to scrambled isomers.

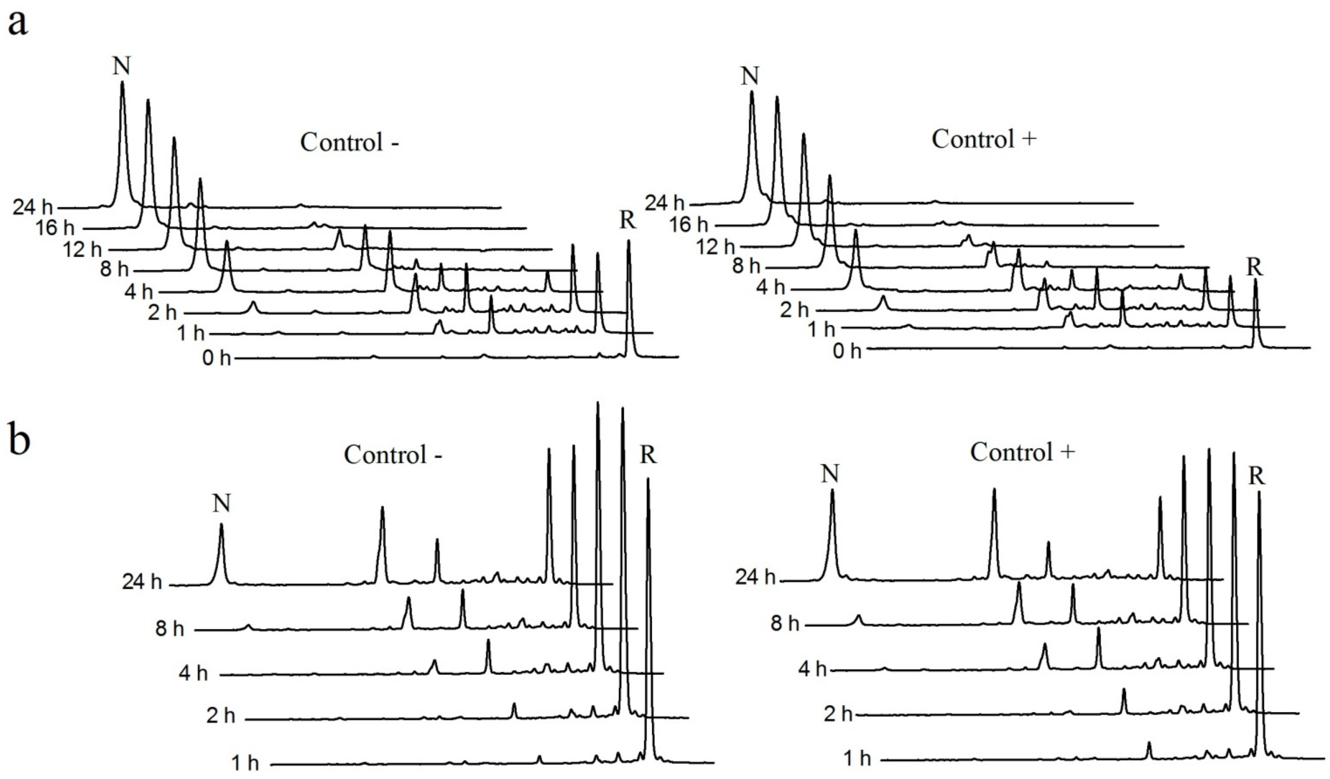

Figure Supplementary S1. . *Inlfuence of temperature and physiological pH on oxidative folding kinetics of NvCI* a) The folding reactions were performed in 0.1 M Tris-HCl (pH 7.4) in the absence (Control -) and presence of 0.25 mM 2-mercaptoethanol (Control +). b) The folding reactions were performed on ice, in 0.1 M Tris-HCl buffer (pH 8.4) in the absence (Control -) and presence of 0.25 mM 2-mercaptoethanol (Control +). Intermediates were acid-trapped at the noted times and analyzed by RP-HPLC. Retention times of the native (N) and fully reduced/unfolded (R) are indicated.

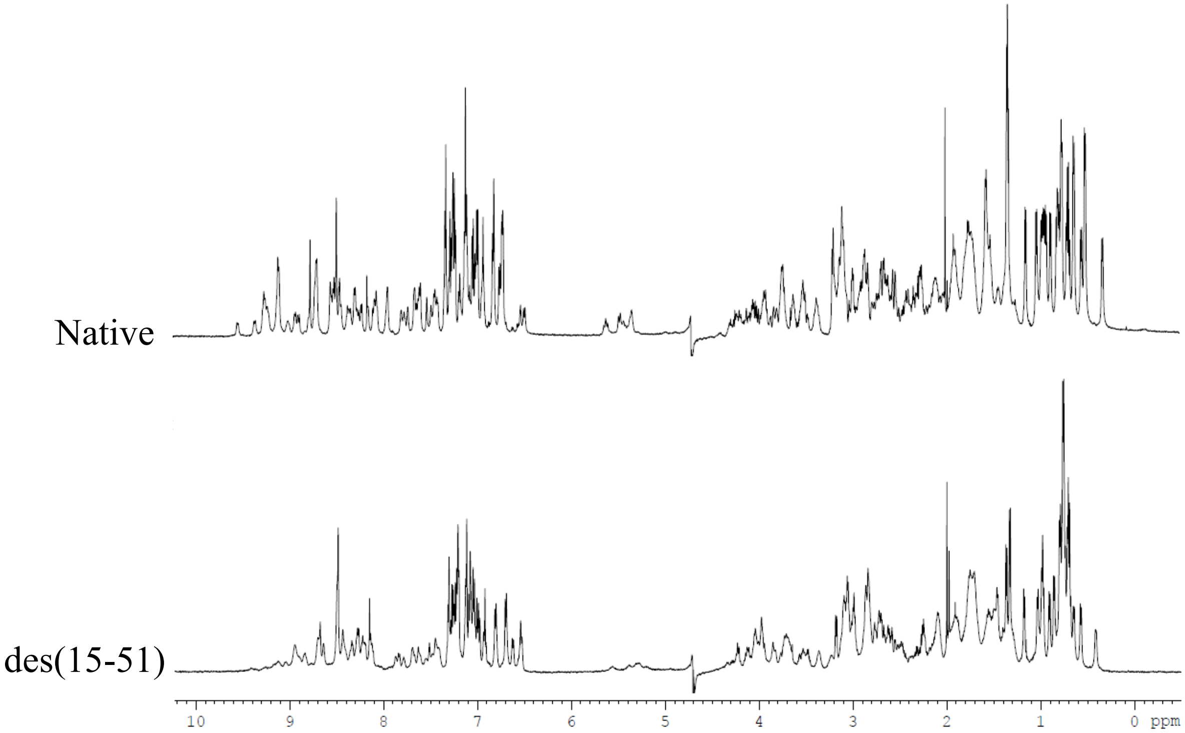

Figure Supplementary S2. *^{1}H NMR analyses of native NvCI and des(15-51)*. Monodimensional spectra were recorded in a Bruker AMX 600-MHz spectrometer at 25 °C. Samples were prepared at 1.5 mg/ml in H_2O/D_2O (9:1 ratio, v/v) at pH 2.0.

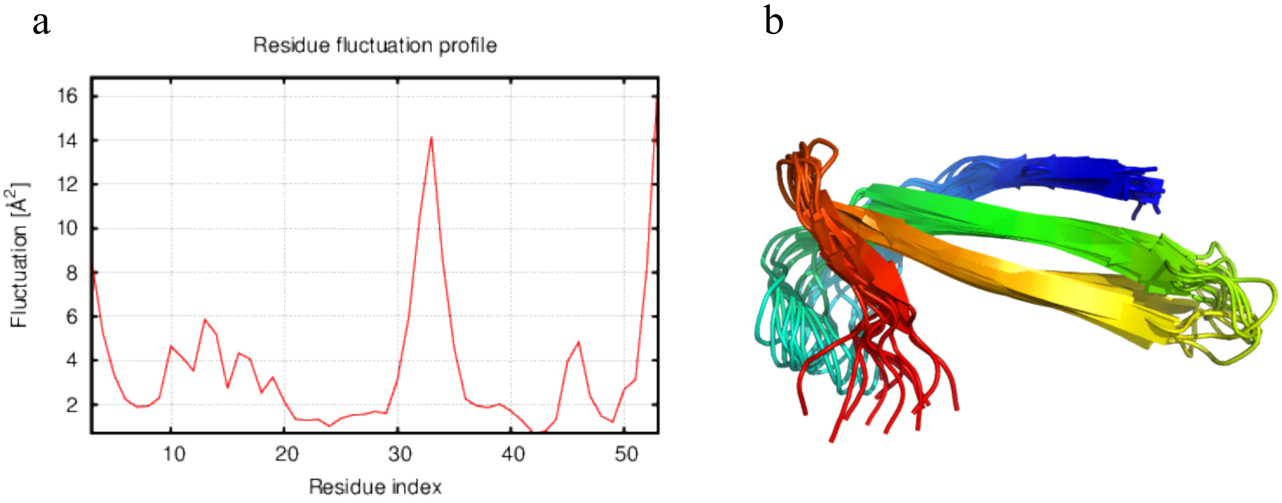

Figure Supplementary S3. Analysis of the conformational flexibility of isolated NvCI using the CABS-FLEX algorithm. a) Residue conformational fluctuation profile. b) Ten most populated conformers in the simulation.

References

1 Chang, J. & Ballatore, A. The structure of denatured bovine pancreatic trypsin inhibitor (BPTI). *FEBS Lett* **473**, 183-187, doi:S0014579300015155 [pii] (2000).

2 Chang, J. Y., Li, L., Canals, F. & Aviles, F. X. The unfolding pathway and conformational stability of potato carboxypeptidase inhibitor. *J Biol Chem* **275**, 14205-14211, doi:275/19/14205 [pii] (2000).

3 Bulychev, A. & Chang, J. Y. Unfolding of hirudin characterized by the composition of denatured scrambled isomers. *J Protein Chem* **18**, 771-778 (1999).

4 Chang, J. Y. & Li, L. The disulfide structure of denatured epidermal growth factor: preparation of scrambled disulfide isomers. *J Protein Chem* **21**, 203-213 (2002).

5 Chang, J. Y., Marki, W. & Lai, P. H. Analysis of the extent of unfolding of denatured insulin-like growth factor. *Protein Sci* **8**, 1463-1468, doi:10.1110/ps.8.7.1463 (1999).
